# Wellens Syndrome in a 28-Year-Old With Pleuritic Chest Pain

**DOI:** 10.7759/cureus.65438

**Published:** 2024-07-26

**Authors:** Tucker Ledo, James Espinosa, Alan Lucerna

**Affiliations:** 1 Emergency Medicine, Jefferson Health, Stratford, USA

**Keywords:** st-elevation mi, stemi, pleuritic chest pain, emergency department management of wellens syndrome, pleuritic chest pain in myocardial infarction, myocardial infarction, wellens syndrome

## Abstract

We report a case of a 28-year-old African American male with months of diffuse, pleuritic, intermittent, non-exertional chest pain with elevated troponin and an ECG showing Wellens syndrome; he was found to have myocardial infarction with 80% proximal left anterior descending (LAD) coronary artery occlusion and 100% distal LAD occlusion. This patient's age and symptoms were not typical for cardiac ischemia, although the ECG was typical. Identification and proper management of Wellens syndrome rely on familiarity with its ECG patterns. Other information such as age, cardiac risk factors, chest pain with exertion and at rest, and elevated troponin are all helpful supplemental information, but as shown in this case report, presentations may vary. This case report demonstrates the importance of having a low threshold of suspicion for Wellens syndrome when faced with indicative ECG abnormalities, despite a patient's history of present illness and physical exam being inconsistent with typical presentations of a patient with cardiac ischemia.

## Introduction

In 1982, H. J. J. Wellens first described the two characteristic ECG patterns that were associated with the stenosis of the proximal left anterior descending coronary artery [1]. Prompt identification of these ECG patterns by emergency medicine physicians is important as a delay in the definitive management of acute coronary syndromes with percutaneous intervention leads to increased mortality [2].

Wellens syndrome is generally known to present clinically like other acute coronary syndromes such as with angina/exertional chest pain [3,4]. However, some variations arise in Wellens syndrome's clinical presentation, demographics, and risk factors.

Here, we will discuss those elements in the context of a 28-year-old male who presented with pleuritic chest pain and was diagnosed with myocardial infarction requiring percutaneous coronary intervention (PCI). The symptoms this patient presented with are not typical for cardiac ischemia and may lead the physician to improperly manage the patient and miss the diagnosis of Wellens Syndrome. This article was previously presented as a poster at the 2024 Rowan 28th Annual Research Day on May 2, 2024.

## Case presentation

A 28-year-old male presented to the emergency department (ED) with a complaint of four to five months of intermittent, diffuse chest pain that lasted for two to five minutes, was worse with deep breaths, was non-exertional, and improved when stretching out his chest wall and especially with drinking water. His last episode occurred while in the shower and was three and a half hours prior to his presentation to the ED. He had no past medical history. He had not been lifting anything heavy recently and denied any recent trauma to his chest wall. He claimed that the pain occurred up to several times per day and seemingly randomly such as while in the shower, while at rest, and while driving. He denied any recent illnesses, recent long-distance travel, recent procedures, any history of blood clots, or having undergone hormone therapy. He denied any known family history of sudden cardiac death but believed his grandfather may have died of a heart attack. He denied any drug use besides smoking marijuana weekly.

The patient's vital signs at presentation were as follows: blood pressure 105/55 mmHg, heart rate 66 beats per minute, temperature 98.3°F, respiratory rate 20 breaths per minute, and he was not in any discomfort.

**Figure 1:**
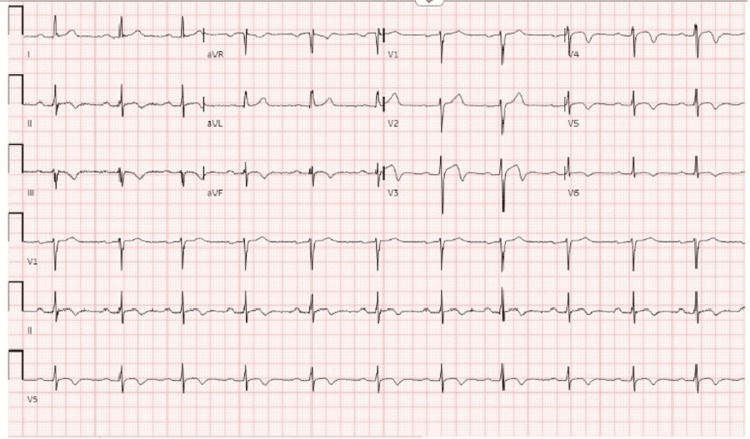
Wellens syndrome ECG ECG showing biphasic T-wave in V3 and V4 characteristic of Wellens Syndrome with T wave inversions in V3-V6, II, III, and aVF representing Wellens’ Type A.

A physical exam showed a healthy-appearing male without chest wall tenderness, rubs, gallops, or murmurs. The lungs were clear to auscultation bilaterally. The ECG showed normal sinus rhythm, had a prolonged PR interval indicative of first-degree atrioventricular block, and showed biphasic T-wave in V3 and V4 characteristic of type A Wellens syndrome along with T wave inversions in V3-V6, II, III, and aVF (Figure 1). His complete blood count and his basic metabolic panel were within normal limits except for an elevated platelet count of 526 B/L. His urine toxicology was negative. His sedimentation rate and hepatic function panel were within normal limits. Abnormal values included high sensitivity troponin of 220 ng/L (three hours after the last episode of chest pain) and a repeat high-sensitivity troponin of 182 ng/L taken two hours after the first troponin was drawn. Total cholesterol was 225mg/dL and LDL was 152mg/dL. The chest X-ray was unremarkable. Cardiology was consulted over the phone and they expressed concern for pericarditis and acute coronary syndrome (ACS). The patient was given oral colchicine, a chewable aspirin tablet of 162 mg, and told to not eat until he was seen by cardiology in the morning.

**Figure 2:**
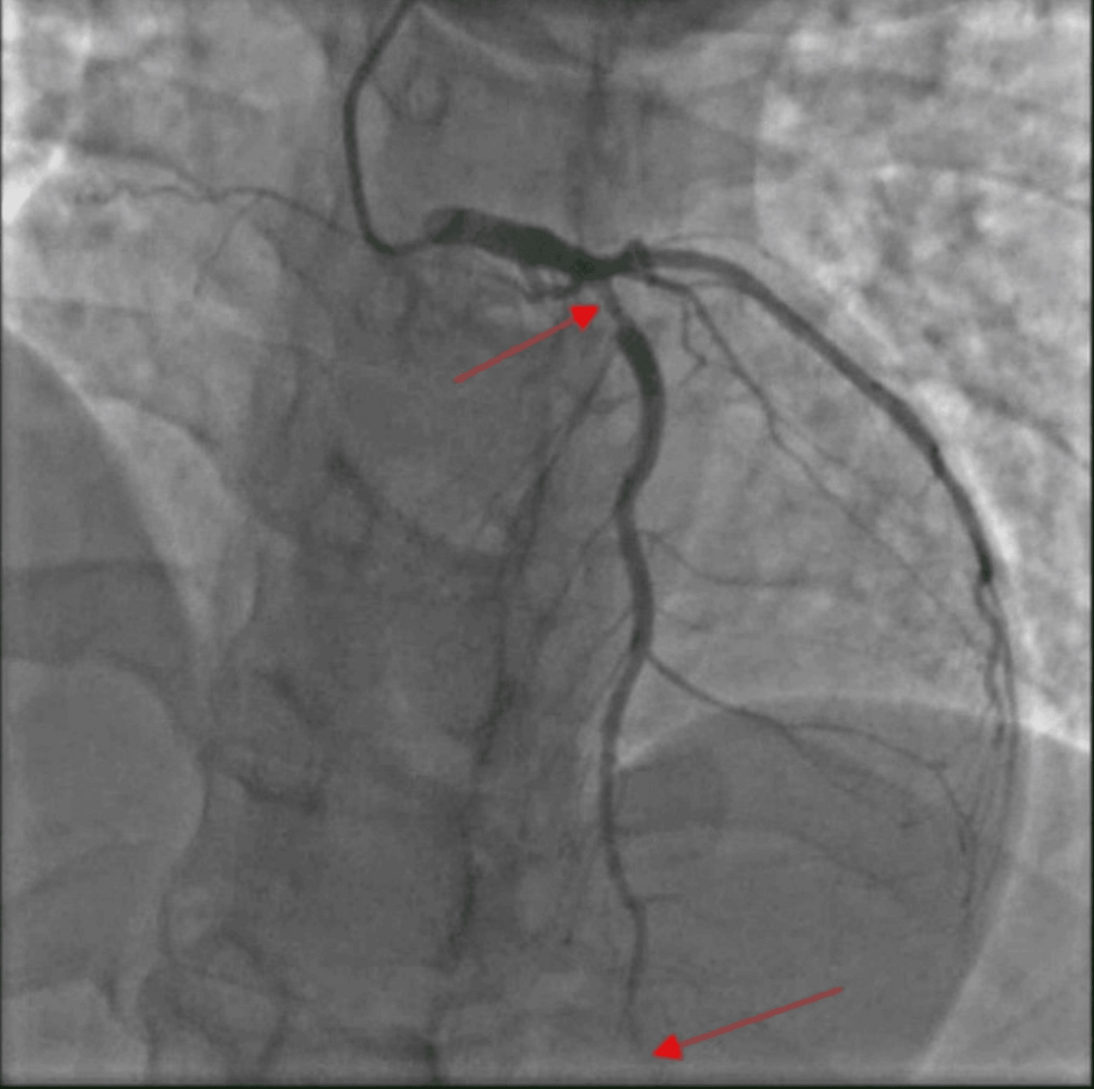
Left heart catheterization in Wellens syndrome The top arrow points to 80% proximal left anterior descending (LAD) occlusion and the bottom arrow to 100% distal LAD occlusion as seen on left heart catheterization.

A computerized tomography angiogram (CTA) scan of the chest was completed with contrast for concern of pericarditis and pulmonary embolism. The results of this study were unremarkable. The patient was admitted to the hospital for presumed pericardial effusion and ACS was ruled out. A transthoracic echocardiogram was unremarkable and without pericardial effusion. The patient then underwent cardiac catheterization, which showed 80% occlusion of the proximal left anterior descending (LAD) coronary artery (Figure 2) and 100% distal occlusion of the LAD (Figure 2). The patient was given clopidogrel with a load of 600 mg and started on a titratable heparin drip without bolus and transferred to another hospital for PCI. Two balloons were used at the proximal LAD site and a drug-eluting stent was placed at the same location. The distal occlusion was not intervened on. The procedure occurred without complications. Following the intervention, the patient was started on low-dose aspirin, clopidogrel, and atorvastatin to be continued outpatient.

## Discussion

The 28-year-old African American man with no known past medical history, later found to have hyperlipidemia, presented with months of diffuse, pleuritic, intermittent, non-exertional chest pain with elevated troponin and the ECG showing Wellens syndrome. He was found to have myocardial infarction with 80% LAD coronary artery occlusion and 100% distal LAD occlusion.

Pathophysiology

Wellens syndrome was first described in 1982 by H. J. J. Wellens as one of two characteristic ECG patterns of T-waves in the precordial leads that have an association with critical stenosis of the proximal LAD coronary artery [1]. The exact mechanism of what causes these specific ECG changes is not certain, but because the ECG changes occur when the patient is pain-free, they are thought to be due to the reperfusion of the ischemic myocardium due to the alleviation of the spasm of the proximal LAD [5-7]. Others have considered that the syndrome may be due to myocardial stunning or myocardial hibernation.

Incidence and presentation of Wellens syndrome

In the largest study available on Wellens syndrome to date, in the 3528 patients who had acute coronary syndrome, the incidence of Wellens syndrome was 5.7% (200 out of 3528). In this same group of patients, those with Wellens syndrome were much less likely to have pre-existing coronary heart disease (39.6% vs. 23%) and previous PCI (19.5% vs. 9%), which is consistent with our patient who had no previous medical history. Wellens syndrome may present on ECG in one of two categories, type A or type B. Type A has biphasic T-waves in leads V2 and V3 that are initially positive and terminally negative, while type B has deeply symmetrical T-wave inversions in leads V2 and V3, often including the other precordial leads as well [8].

Approximately 24% of Wellens cases are type A, and this finding is more specific for Wellens syndrome. The remaining 76% of cases are type B and are less specific; our patient presented with the rarer and more specific form of Wellens syndrome: type A [9].

Uncharacteristically, our patient was 28 years old while, according to Zhou et al., the average age for the occurrence of Wellens syndrome is 63 years old +/- 10.5 years [10]. Our patient also presented with pleuritic chest pain and improvement of pain if he stretched out his chest wall or drank water, all of which are rarely or are never evident in the current data or case reports available today. Each of these are possible detractors from the actual diagnosis and could lead us to believe that this pain was esophageal or from a condition more associated with pleuritis rather than a myocardial infarction.

Laboratory studies, imaging, and management

Due to the suspected pathophysiology of Wellens syndrome, many patients who present do not have elevated cardiac troponin levels. Those patients who are evaluated immediately after an episode of chest pain are more likely to have elevated troponin levels, such as our patient. Current reports indicate that 69% of Wellens syndrome cases present as non-ST-segment elevation myocardial infarction (NSTEMI), while 31% present as unstable angina pectoris (no elevation of troponin) [10].

In Wellens syndrome, early consultation of cardiology/interventional cardiology for left heart catheterization (LHC) is essential to properly identify areas of occlusion and for the greatest survival outcomes. Early PCI is the preferred treatment [10,11]. In this patient, due to the concern for Wellens syndrome, the treatment for ACS ought to have been started as soon as possible in the emergency department.

## Conclusions

Wellens syndrome generally presents clinically similar to NSTEMI or unstable angina but, as demonstrated in the patient in this case report, it can present with atypical symptoms. The patient presented in the report had non-exertional pleuritic chest pain and was significantly younger than the usual demographic of those presenting with acute cardiac ischemia. If the history of the present illness was relied upon for diagnosis rather than proper interpretation of the ECG, the proper diagnosis would have been missed. This case report exemplifies the importance of proper interpretation of the ECG as well as having a low threshold to consider Wellens syndrome despite an atypical presentation. 
